# Comparison of quantitative flow ratio and fractional flow reserve with myocardial perfusion scintigraphy and cardiovascular magnetic resonance as reference standard. A Dan-NICAD substudy

**DOI:** 10.1007/s10554-019-01737-z

**Published:** 2019-11-19

**Authors:** Martin Sejr-Hansen, Jelmer Westra, Simon Winther, Shengxian Tu, Louise Nissen, Lars Gormsen, Steffen E. Petersen, June Ejlersen, Christin Isaksen, Hans Erik Bøtker, Morten Bøttcher, Evald H. Christiansen, Niels Ramsing Holm

**Affiliations:** 1 Department of Cardiology, Aarhus University Hospital, Århus, Denmark; 2 School of Biochemical Engineering, Shanghai Jiao Tong University, Shanghai, China; 3 Department of Cardiology, Hospital Unit West Jutland, Herning, Denmark; 4 Barts Heart Centre, Barts Health NHS Trust, London, UK; 5 William Harvey Research Institute, NIHR Barts Biomedical Research Centre, Queen Mary University of London, London, UK; 6 Department of Nuclear Medicine, Hospital Unit West Jutland, Herning, Denmark; 7 Department of Radiology, Regional Hospital of Silkeborg, Silkeborg, Denmark

**Keywords:** Quantitative flow ratio, Fractional flow reserve, Myocardial perfusion scintigraphy, Cardiovascular magnetic resonance, Stable angina, Myocardial ischemia

## Abstract

Quantitative flow ratio (QFR) and fractional flow reserve (FFR) have not yet been compared head to head with perfusion imaging as reference for myocardial ischemia. We aimed to compare the diagnostic accuracy of QFR and FFR with myocardial perfusion scintigraphy (MPS) or cardiovascular magnetic resonance (CMR) as reference. This study is a predefined post hoc analysis of the Dan-NICAD study (NCT02264717). Patients with suspected coronary artery disease by coronary computed tomography angiography (CCTA) were randomized 1:1 to MPS or CMR and were referred to invasive coronary angiography with FFR and predefined QFR assessment. Paired data with FFR, QFR and MPS or CMR were available for 232 vessels with stenosis in 176 patients. Perfusion defects were detected in 57 vessel territories (25%). For QFR and FFR the diagnostic accuracy was 61% and 57% (p = 0.18) and area under the receiver operating curve was 0.64 vs. 0.58 (p = 0.22). Stenoses with absolute indication for stenting due to diameter stenosis > 90% by visual estimate were not classified as significant by either QFR or MPS/CMR in 21% (7 of 34) of cases. The diagnostic performance of QFR and FFR was similar but modest with MPS or CMR as reference. Comparable performance levels for QFR and FFR are encouraging for this pressure wire-free diagnostic method.

## Introduction

Fractional flow reserve (FFR) and recently also instantaneous wave-free ratio (iFR) are the gold standard indices for invasive functional assessment of coronary artery stenosis [1, 2]. Despite the compelling evidence, FFR remains underused in clinical practice [3, 4]. Quantitative flow ratio (QFR) is a rapid, computed approximation of FFR based on 3D reconstruction of the coronary vessel and estimated contrast flow velocity derived from standard diagnostic invasive coronary angiography (ICA). QFR does not require induction of hyperemia or the use of pressure wires and can be computed within the time of conventional FFR measurement [5–7]. QFR approaches FFR with an overall good diagnostic accuracy (ranging from 80 to 95%) [5, 6, 8–11] and recent studies reported a good agreement between QFR and myocardial perfusion scintigraphy, MPS [1, 5, 12].

Present clinical guidelines recommend FFR or iFR measurement in patients with stable angina pectoris and narrowings with a diameter stenosis (DS) of 40–90% by visual assessment, when no other evidence of ischemia is available [13]. However, even experienced interventional cardiologists tend to overestimate percent DS, leading to inappropriate deferral of wire-based assessment of visual severe lesions [4].

To our knowledge, QFR and FFR have not previously been compared using perfusion imaging as reference for myocardial ischemia. Therefore, we aimed to test the hypothesis that QFR and FFR perform equally when MPS and cardiovascular magnetic resonance (CMR) are used as reference to identify myocardial ischemia in patients with stable angina pectoris. Moreover, as we aimed to assess the potential use of QFR in vessels with “absolute” indication of stenting due to more than 90% stenosis by visual estimate.

## Methods

### Study design

This is a pre-planned substudy and the design of the main Dan-NICAD study was previously published [14]. In short, CCTA is the preferred first-line test in patients with stable chest pain in Denmark. Dan-NICAD was designed to compare the diagnostic performance of SPECT and CMR as second line test after identification of suspected obstructive CAD (50–100% DS) by CCTA in symptomatic patients. All vessels in the Dan-NICAD study that were successfully assessed by FFR or had operator assessed severity of at least 90% DS were included in this substudy and underwent QFR analysis. QFR analysis reported in the WIFI II substudy were included if applicable [6].

### Non-invasive imaging

A summed stress score (SSS), a summed rest score (SRS), and a summed different score (SDS) were generated for MPS. An abnormal MPS scan was defined as (i) an SDS ≥ 4 involving ≥ 2 contiguous segments, (ii) an SRS ≥ 4 involving ≥ 2 contiguous segments, and (iii) a combination of (i) and (ii).

For CMR, an abnormal scan was defined as subendocardial or transmural changes by stress imaging or irreversible defects in ≥ 2 contiguous segments by late gadolinium enhancement imaging. Full description of MPS and CMR analysis was reported in the Dan-NICAD papers [14, 15]. CMR core-lab readings were performed in an experienced core lab (William Harvey Research Institute, Queen Mary University of London, London, UK) while MPS Images were analyzed by a different independent core lab (Department of Nuclear Medicine, Aarhus University Hospital, Aarhus, Denmark).

### Invasive coronary angiography and fractional flow reserve

FFR was attempted in all coronary segments with a diameter > 2.0 mm and visual 30–90% DS. Inconsistencies were countered by using validated pressure wires (Volcano or Abbot-St-Jude), sole use of IV adenosine (140 μg/min/kg), and by core-lab FFR reading of pressure traces (Interventional Coronary Imaging Core Laboratory; Aarhus University Hospital, Denmark). Two-dimensional quantitative coronary angiography (2D-QCA) was performed by an independent core lab (ClinFact, Leiden, The Netherlands). A DS of 50% or more was used as a diagnostic cutoff value. Using the American Hearth Associations 17 segment model, segment 1, 2, 5–7 and 11 where defined as proximal/mid. Remaining segments were defined as distal. We refer to the Dan-NICAD papers for further details [14, 15].

### QFR computation

**Fig. 1:**
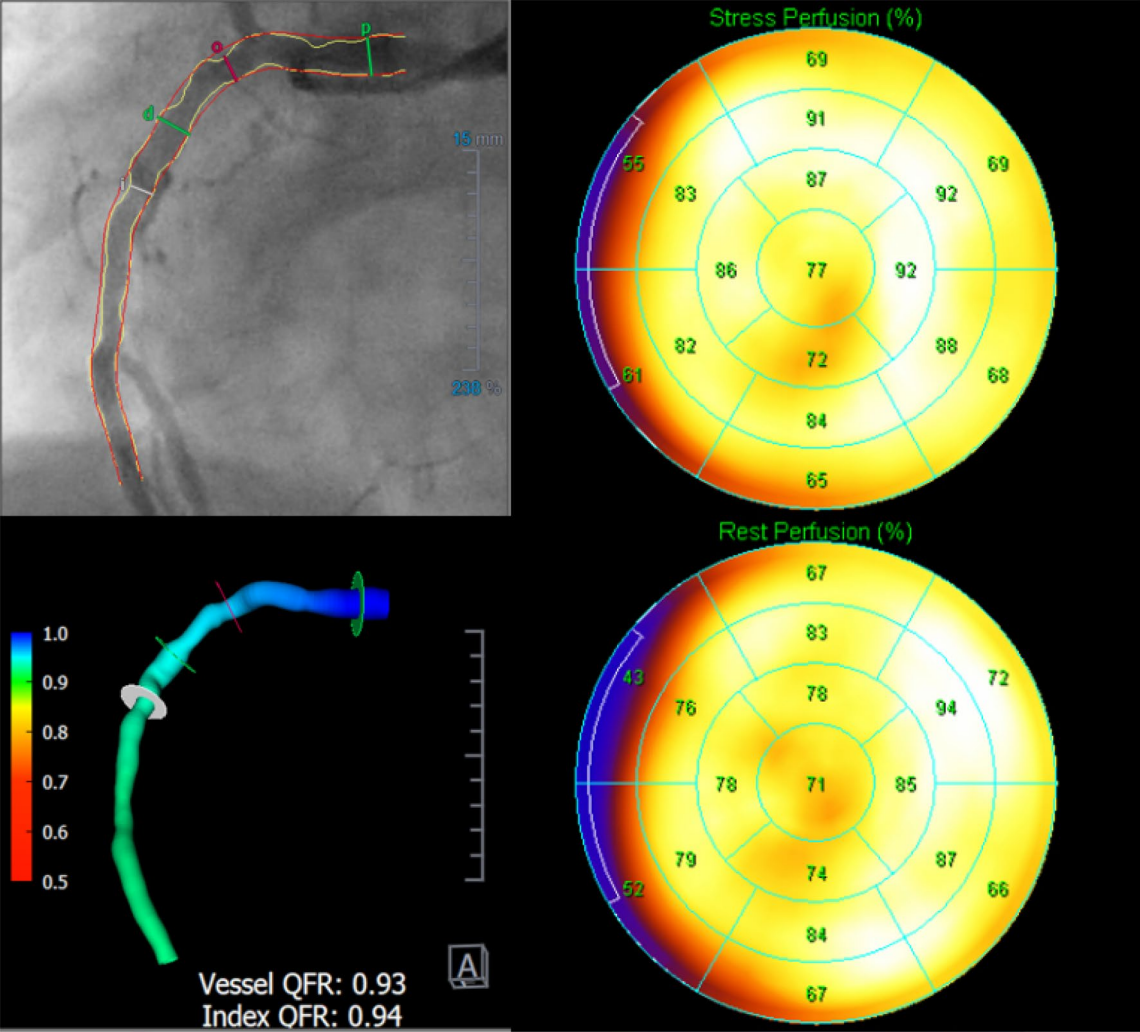
Study procedure. Example of study procedure showing a vessel visually estimated as > 90% stenosis with normal MPS assessment. Right side represents the MPS results, upper left is QCA, lower left show QFR results in a flow distribution map of the vessel. *MPS* myocardial perfusion scintigraphy, *QCA* quantitative coronary angiography, *QFR* quantitative flow ratio

The QFR methodology was previously described [5]. QFR was computed off-line using the QAngio XA 3D software QFR (Medis Medical Imaging B.V., The Netherlands) in a core-lab by blinded investigators. A diagnostic cutoff value of ≤ 0.80 was used. Further details on QFR computation in the Dan-NICAD study are found in the WIFI-II report [6]. See Fig. 1 for analysis example.

### Statistical analysis

Continuous variables are presented as mean ± SD or median with interquartile range as appropriate. Categorical variables are presented as numbers and percentage. Normal distribution was checked using QQ-plots and the Shapiro–Wilk test. The diagnostic accuracies of QFR and FFR are presented with sensitivity, specificity, negative predictive value, and positive predictive value, and were compared using McNemar’s test and generalized score statistics. Comparison between the area under receiver operating curve (AUC) of QFR and FFR was made using the DeLong method. Optimal cutoff values were found using Youden’s method.

## Results

**Fig. 2:**
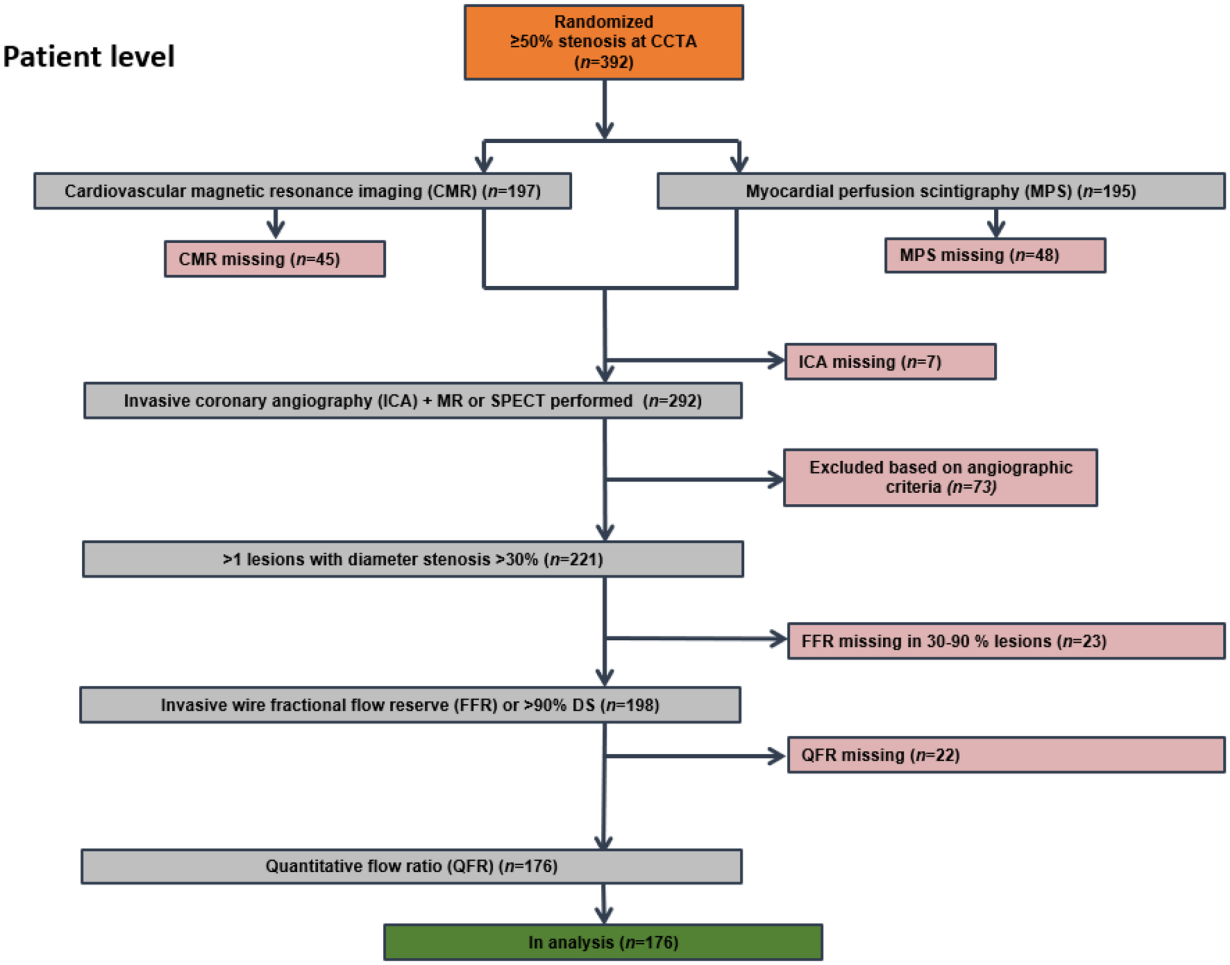
Patient level flowchart. n represents the number of included patients. *QFR* quantitative flow ratio, *FFR* fractional flow reserve, *CMR* cardiovascular magnetic resonance imaging, *MPS* myocardial perfusion scan, *ICA*  invasive coronary angiography, *DS* diameter stenosis, *CCTA* coronary computed tomography angiography

**Fig. 3:**
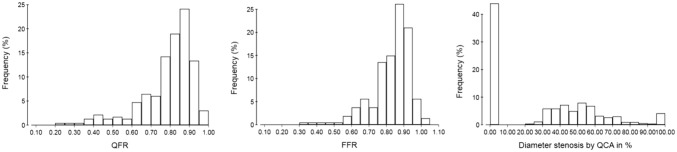
Distribution of QFR, FFR and diameter stenosis. Histograms of QFR (left), FFR (middle) and diameter stenosis per 2D-QCA (right). *QFR* quantitative flow ratio, *FFR* fractional flow reserve, *QCA* quantitative coronary angiography

A total of 392 patients with CCTA-identified coronary artery disease were assessed by myocardial perfusion imaging and randomly assigned to MPS (n = 195) or CMR (n = 197). Complete paired dataset with perfusion imaging, QFR, and FFR were available for 176 patients (90 for MPS and 86 for CMR) and 232 stenosis/perfusion territories (Fig. 1). Baseline demographics, clinical characteristics and study flow chart are listed in Table 1 and Fig. 2. Distribution of key variables is visualized in Fig. 3. For lesions with paired assessment of QFR and FFR, median QFR was 0.84 (IQR 0.77–0.89) and median FFR was 0.85 (IQR 0.78–0.91).

**Table 1:** Patient characteristics

Patient characteristics	(n = 176)
Female gender, n (%)	57 (32)
Age (years), mean ± SD	61 ± 8.1
BMI, mean ± SD	27 ± 3.9
Current smoker, n (%)	34 (19)
Antihypertensive treatment, n (%)	50 (28)
Lipid-lowering treatment, n (%)	20 (11)
Diabetes, n (%)	14 (8)
Family history of early CAD, n (%)	66 (38)
Symptoms, n (%)	
Typical angina	54 (31)
Atypical angina	61 (35)
Non-anginal chest pain	28 (16)
Dyspnoea or arrhythmia	33 (19)

### Patient level diagnostic performance

A total of 44 (25%) patients had perfusion defects on either MPS (19) or CMR (25). A total of 4 (2%) patients had resting perfusions abnormalities by CMR while 6 (3%) had resting perfusion abnormalities by MPS. QFR and FFR identified at least one flow-limiting stenosis in 85 (48%) and 81 (46%) patients, respectively. The overall diagnostic performance was similar for QFR and FFR with diagnostic accuracy of 59% (95% CI 51–66) and 60% (95% CI 52–67), (p = 0.88); AUC 60% (95% CI 52–69) and 66% (95% CI 58–73), (p = 0.23); sensitivity 64% (95% CI 49–78) and 77% (95% CI 62–89), (p = 0.15); specificity 57% (95% CI 48–65) and 54% (95% CI 45–63), (p = 0.69); positive predictive value was 33% (95% CI 23–44) and 36% (95% CI 26–46), (p = 0.39) and negative predictive value was 82% (95% CI 73–90) and 88% (95% CI 79–94), (p = 0.13) using MPS or CMR as reference standard.

### Lesion level diagnostic performance

**Fig. 4:**
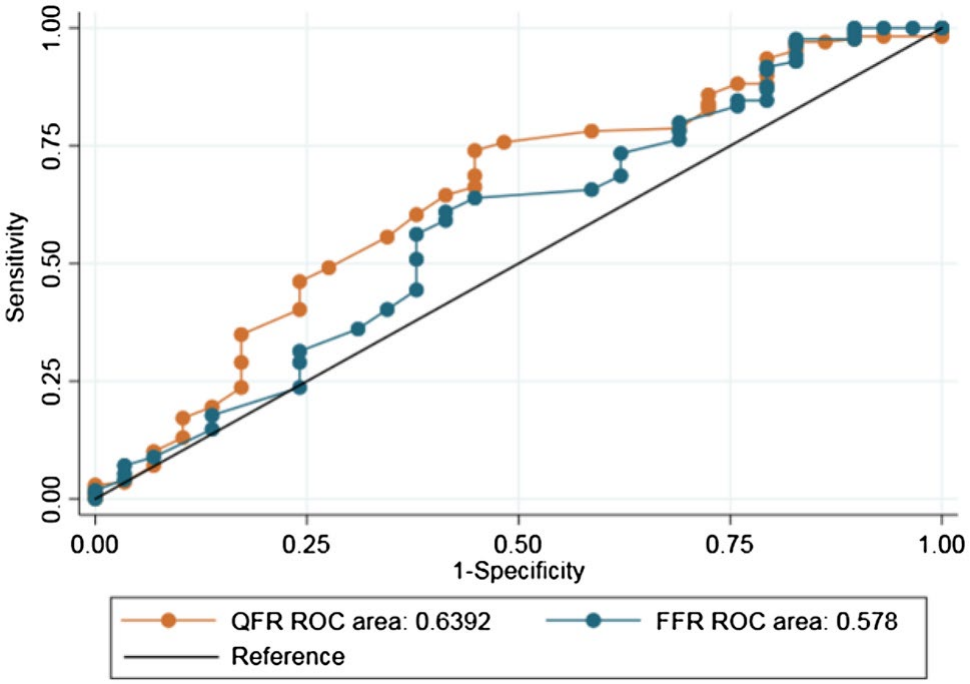
ROC curve analysis. Comparison of ROC curves for QFR and FFR with non-invasive imaging as reference. *ROC* receiver-operating characteristics, *QFR* quantitative flow ratio, *FFR* fractional flow reserve

**Fig. 5:**
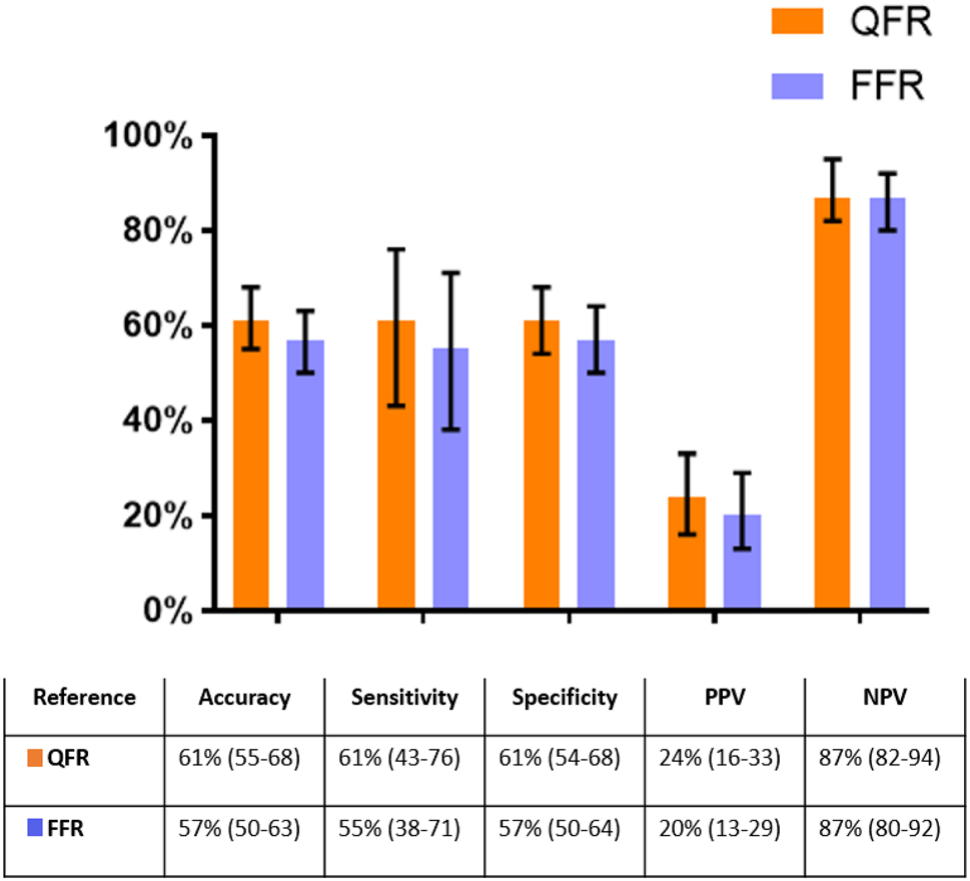
Lesion-level diagnostic performance of QFR and FFR. Diagnostic performance of QFR and FFR with non-invasive imaging as reference presented as percentage with 95% confidence intervals. Results are numerically summarized in the table. *QFR* quantitative flow ratio, *FFR* fractional flow reserve, *PPV* positive predictive value, *NPV* negative predictive value

Perfusion defects were detected in 57 (25%) of 232 vessel territories. QFR and FFR identified 98 (42%) and 131 (56%) vessels with hemodynamically significant lesions. For the 232 vessels available for analysis, 36% were located in proximal/mid segment and 64% in distal segments.QFR and FFR showed no difference in diagnostic performance parameters as shown in Figs. 4 and 5.

### Diagnostic performance of 2D-QCA

**Fig. 6:**
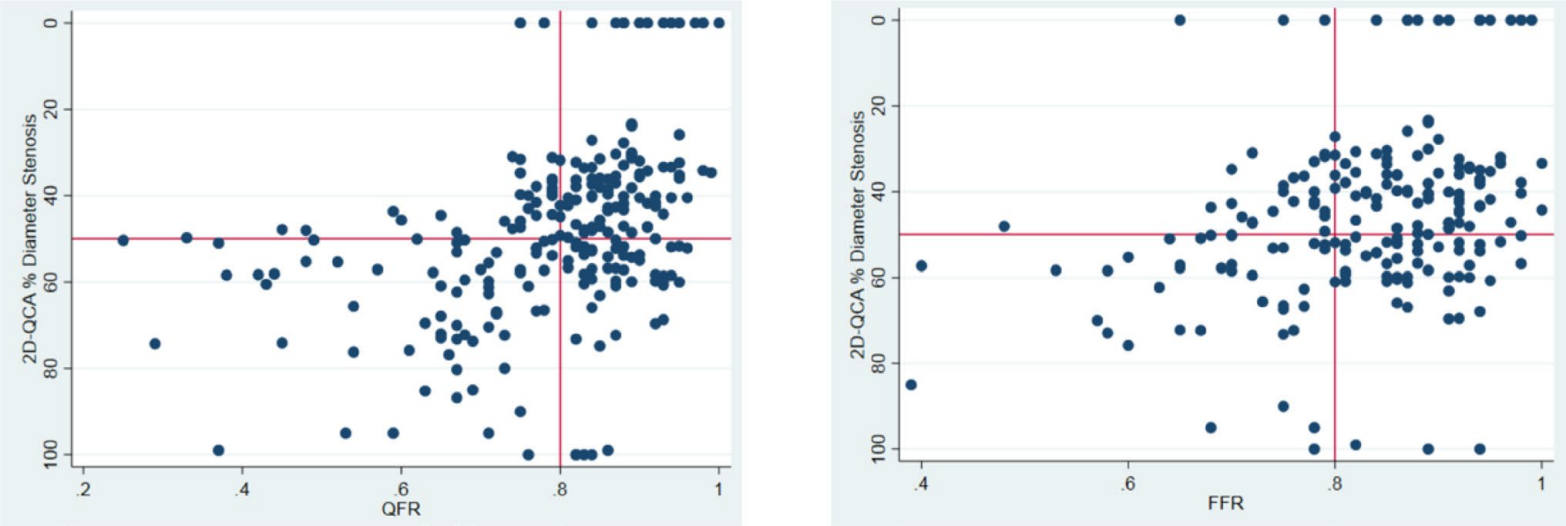
Scatterplots of QFR and FFR in relation to 2D-QCA. Comparison of QFR and FFR to 2D-QCA respectively. Red lines indicate cut-off values. *QFR* quantitative flow ratio, *FFR* fractional flow reserve, *2D-QCA* two-dimensional quantitative coronary angiography

2D-QCA correlated poorly with QFR (Rho: 0.46) and FFR (Rho: 0.30) (Fig. 6). A total of 116 (50%) lesions were deemed hemodynamically significant by QCA. Diagnostic accuracy was 55% (95% CI 49–62), AUC 59% (95% CI 51–68), sensitivity 65% (95% CI 49–80), specificity 53% (95% CI 46–60), positive predictive value 22% (95% CI 15–30), and negative predictive value was 89% (95% CI 82–94) using MPS or CMR as reference standard.

### Optimal cut-off values

The optimal cutoff values for QFR and FFR to identify myocardial ischemia were ≤ 0.78 and ≤ 0.82 using MPS and CMR as reference for myocardial ischemia.

### Diameter stenosis: visual estimation compared to 2D-QCA

In the 597 vessels where DS% were assessed by 2D-QCA and eyeballing, the mean overestimation of DS% by visual estimation was 14% ± 3.5. Overestimation of DS% by operator eyeballing was in the left anterior descending artery 14% ± 16 (n = 471), the left circumflex artery 5% ± 14 (p = < 0.01, n = 60), and in the right coronary artery 5% ± 15 (p = 0.01, n = 66).

### Stenosis with more than 90% DS by physicians visual estimate

**Fig. 7:**
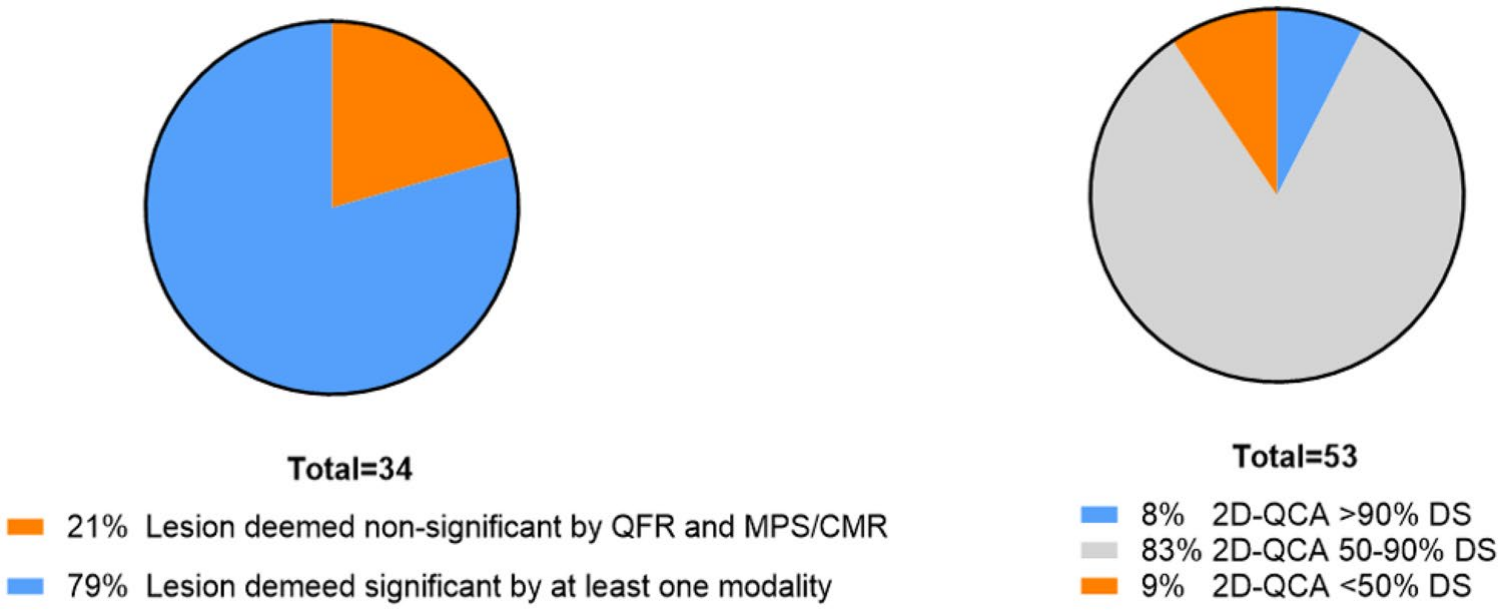
Subanalysis of lesions deemed too severe for FFR by visual estimation. Pie-chart showing subanalysis of lesions visually estimated to be DS > 90%. 34 of 53 lesions were applicable for QFR analysis. *MPS* myocardial perfusion scan, *QCA* quantitative coronary angiography, *QFR* quantitative flow ratio, *CMR* cardiovascular magnetic resonance, *DS* diameter stenosis

FFR was not applicable in 53 patients due to severe stenosis visually estimated ≥ 90% DS. QFR was computed in 34 (64%) of these lesions with a mean DS Medis Medical Imaging B.V. (2D-QCA) of 68% ± 17 and mean QFR of 0.64 ± 0.18. A total of 9 (17%) perfusion defects were identified by MPS (2) or CMR (7) in this subgroup. Compared to visual estimation, used to classify stenosis too severe for FFR measurement, QFR showed a better diagnostic accuracy [41% (95% CI 24–49) vs. 26% (95% CI 11–42) p = 0.04] using MPS or CMR as reference. A total of 21% of lesions were non-significant by MPS/CMR or by QFR while 8% had a DS% > 90% by 2D-QCA (Fig. 7).

## Discussion

This is the first study to compare QFR and FFR to perfusion imaging as reference test for myocardial ischemia. The two modalities showed a similar but limited ability to predict myocardial perfusion defects as identified by MPS or CMR. Additionally, we showed that QFR may be useful for appropriate evaluation of visually estimated severe lesions not applicable for FFR measurement.

The current findings add to the existing knowledge that QFR agrees with wire-based FFR in the majority of patients in head to head comparisons [5, 6, 11, 16]. We found a low sensitivity of QFR and FFR when CMR and MPS were used as reference. This may be explained by several factors. First, FFR may produce false negatives related to increased microvascular resistance leading to a decreased flow velocity that could increase FFR [17]. Recent work from Mejia-Renteria et al. indicates that this may also be the case for QFR [18]. Second, FFR may produce false positives related to high coronary flow reserve. The discordance between FFR, MPS, and CMR may be further characterized with invasive CFR and IMR assessment, as planned in future studies [19]. Further, in vessels with proximal or mid-segment lesions, non visualized stenosis distally to the location of the pressure-wire may influence the final FFR and QFR measurements.

Previous work from Cook et al. correlated high coronary flow reserve values to cases with positive FFR but negative instantaneous flow ratio (iFR), questioning wether the low FFR values reflect flow-limiting stenosis [20]. The recent MR-INFORM study showed that a CMR-guided revascularization strategy led to less revascularization while being non-inferior to an FFR-guided strategy by a composite endpoint of death, myocardial infarction and target vessel revascularization [21]. The overall low classification agreement between QFR and FFR with MPS and CMR may thus reflect the cumbersome progress of identifying myocardial ischemia. 2D-QCA showed a modest classification agreement with MPS and CMR while the correlation to FFR and QFR was poor in line with previous studies. No present available stand-alone modality can identify and measure all aspects related to the development of myocardial ischemia. Thus, the differences in measuring myocardial perfusion or solely the contribution of epicardial disease should be acknowledged. The current revascularization guidelines are focused on epicardial lesions and it is therefore promising that the diagnostic performance of QFR is comparable to FFR when using myocardial perfusion as reference standard [22]. Importantly, FFR was itself initially validated using a cascade of non-invasive modalities, including MPS, as reference standard for myocardial ischemia [23, 24].

In contrast with the presented findings, Smits and colleagues found a high diagnostic accuracy for QFR to detect ischemia based on MPS (90% accuracy) [12]. Smits et al. included 85 patients and 255 arteries with milder disease (mean DS 11.8% and mean QFR 0.96) compared to this study (mean DS 30.4% and mean QFR 0.81), which may attribute to the observed disparity.

The presented visual overestimation of DS% compared to measurement by 2D-QCA is in line with prior findings [4]. We found that 21% of stenosis with > 90% DS did not produce ischemia as assessed by non-invasive imaging nor by QFR, despite a direct indication for revascularization. Thus, an FFR based revascularization strategy including visual estimation may be sensitive to the visual overestimation of stenosis severity and could lead to unnecessary revascularization. For severely stenosed vessels, the low success rate and risks associated with advancing a pressure wire had guidelines cautioning the use of FFR in severe stenosis [22, 24]. If our findings are confirmed, the wire-free nature of QFR could allow for assessment of lesions exceeding 90% DS.

A recent study by Tebaldi and colleagues demonstrated that eyeballing remains a major limiting factor for FFR adoption [25]. Our data indicates additional value of QFR in severe lesions that could supplement an FFR-based revascularization strategy at this time and adds to the mounting evidence on QFR’s potential as a diagnostic tool for functional evaluation of intermediary coronary artery stenosis pending further validation in randomized outcome trials [5–7, 10, 11].

## Limitations

The results should be interpreted keeping in mind the limited number of perfusion defects detected by MPS and CMR. However, the study set-up reflects a regular local clinical referral-strategy, in accordance with the NICE and ESC guidelines, where CCTA is performed as first-line test. The distribution of lesion severity assessed by FFR was comparable to major all-comer studies [26, 27]. Despite that both modalities reflect myocardial perfusion, CMR and MPS are not identical, and do not have the same diagnostic characteristics, hence combining them as reference standard could affect inference of our results. Since QFR and FFR only disagree in 10–20% of all cases, the present sample was not adequately sized for a sub-analysis of patients with diagnostic disagreement. Our population of lesions in the subgroup with DS of at least 90% was limited and the findings are thus solely hypothesis generating.

## Perspectives

Our results suggest that QFR could be an alternative to FFR in assessing the severity of intermediate stenosis. QFR may further emerge a diagnostic method for assessing severe stenosis otherwise considered significant solely based on visual assessment of the angiographic appearance. Due to the poor agreement between MPS/CMR and FFR/QFR in this low to intermediate risk population, more data is required to support the NICE guidelines of using perfusion imaging as second line test.

## Conclusion

Diagnostic performance of QFR and FFR was similar but modest with MPS and CMR as reference. The comparable performance levels by QFR and FFR are encouraging for pressure wire-free, functional lesion evaluation. QFR may further be useful for evaluation of severe stenosis where FFR measurement is deferred.
